# An Efficient Lagrangean Relaxation-based Object Tracking Algorithm in Wireless Sensor Networks

**DOI:** 10.3390/s100908101

**Published:** 2010-08-27

**Authors:** Frank Yeong-Sung Lin, Cheng-Ta Lee

**Affiliations:** Department of Information Management, National Taiwan University, No. 1, Sec. 4, Roosevelt Rd., Taipei City 106, Taiwan; E-Mail: yslin@im.ntu.edu.tw (F.Y.-S.L.)

**Keywords:** wireless sensor networks, object tracking, Lagrangean relaxation

## Abstract

In this paper we propose an energy-efficient object tracking algorithm in wireless sensor networks (WSNs). Such sensor networks have to be designed to achieve energy-efficient object tracking for any given arbitrary topology. We consider in particular the bi-directional moving objects with given frequencies for each pair of sensor nodes and link transmission cost. This problem is formulated as a 0/1 integer-programming problem. A Lagrangean relaxation-based (LR-based) heuristic algorithm is proposed for solving the optimization problem. Experimental results showed that the proposed algorithm achieves near optimization in energy-efficient object tracking. Furthermore, the algorithm is very efficient and scalable in terms of the solution time.

## 1. Introduction

The rapid growth in sensor technology and wireless communication has resulted in the development of wireless sensor networks (WSNs). Their main features are low cost and wireless communication capability. They consist of several sensors, sink nodes, and back-end systems. These sensors work in coordination to collect physical information from the sensor field, and they can process and forward the information to the sink nodes. Finally, the back-ends can obtain global views according to the information provided by the sink nodes [1,2].

Object tracking is a key application issue of WSNs which is widely deployed for military and wildlife animal tracking purposes. Object tracking wireless sensor networks have two critical operations [3–5]. One is monitoring. Sensor nodes are required to detect and track the moving states of mobile object. The other is reporting. The nodes sensing the object need to report their discoveries to the sink. These two operations are interleaved during the entire object tracking process. We assume that the moving frequencies of the sensor field are not uniformly distributed. For example, the moving frequencies of wild animals in a wildlife protective zone are not uniform, because animals usually move in their customary paths.

Our focus in prior studies [6–8], has been on developing strategies for reducing the energy consumption in reporting operations. In [6], Kung, *et al.* proposed a scalable tracking method for sensor tracking systems using network sensors called STUNs. The tracking system is a scalable tracking architecture that employs a hierarchical structure to allow the system to handle a large number of tracked objects. Furthermore, authors proposed a drain-and-balance (DAB) method to construct a STUN’s hierarchical structure based on expected properties of the object movement patterns such as the frequency of object movements over a monitoring region. In [7,8], Lin, *et al.* proposed two message-pruning tree structures called DAT and Z-DAT for object tracking. The two methods are used to construct an object tracking tree for reducing the communication cost of location updating. The Z-DAT approach tries to divide the sensing area into square-like zones and recursively combine these zones into a tree.

This study is an extension of the work in [6–8]. The prior studies are expanded to encompass energy-efficient object tracking in wireless sensor networks. We focus on the problem of constructing an energy-efficient wireless sensor network for object tracking services using the object tracking tree. This tree proposes a data aggregation model for object tracking [9–12]. Therefore, we were motivated to propose a heuristic strategy to cope with the problem. With a given arbitrary sensor network topology, we consider in particular the case of bi-directional moving objects with given frequencies for each pair of sensor nodes and link transmission cost. The total communication cost can be computed and minimized by the object tracking tree.

In this paper, we formulate the problem as a 0/1 integer-programming problem where the objective function is to minimize the total communication cost subject to routing, tree, and variable-transformation constraints. The object tracking tree is a weighted spanning graph of given sensor and communication nodes. The tree is used to minimize total communication cost. Therefore, constructing the object tracking tree is an NP-complete problem [13]. A method called Lagrangean relaxation has been successfully adopted to solve many famous NP-complete problems [14,15]. To fulfill the timing and the quality requirements of the optimal decisions, the Lagrangean relaxation method is used. We use the LR-based heuristic algorithm to solve the problem and obtain a primal feasible solution. In the further experiments, the proposed object tracking algorithm is expected to be efficient and effective in dealing with the complicated optimization problem.

Compared to [6–8], this study differs from the prior works on two points. First, we consider the case of bi-directional moving objects with given frequencies for each pair of sensor nodes and link transmission cost. Second, we present a LR mathematical model to describe the optimization problem and propose LR-based heuristic algorithm to solve the problem.

The rest of this paper is organized as follows. The problem and mathematical model are described in Sections 2 and 3, respectively. Additionally, the solution approach is presented in Section 4. Furthermore, the computational results are discussed in Section 5. Finally, our conclusions is presented in Section 6.

## 2. Problem Description

Our approach uses a hierarchical object tracking tree to record information about presence of the object and keep this information up to date [6]. The leaves of the tree are sensor nodes, and they are required to detect and track the moving states of mobile objects. The other nodes are communication nodes, and the information about presence of the detected objects is stored at these communication nodes. Each communication node stores in particular the set of objects that was detected jointly by its descendants. The set is called the detected set. For example, the detected set of a sensor at a leaf node consists of the objects within the detection range of sensor while the detected set of sink node contains all objects presented in the sensor field.

**Figure 1.**
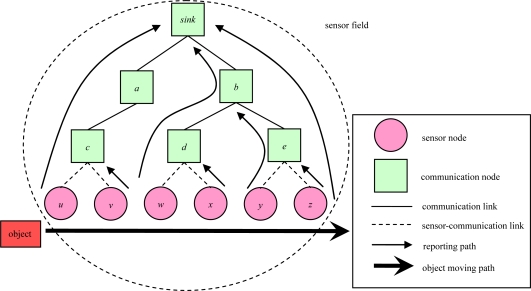
An example of object tracking.

Figure 1 illustrates an object tracking scenario. Sensor *u* will detect the object and deliver the object’s location information to the sink node when the object enters the sensor field, and sensor *v* will only forward the new location information to communication node *c* when object moves from sensor *u* to sensor *v*. This scenario can be performed through the entire sensor field. Finally, sensor *z* will forward the leaving information to sink node when object leaves sensor field from sensor *z*. The problem is solved in the planning stage.

The problem of energy-efficient object tracking in WSNs is modeled as a graph, *G*(*V*,*L*), where *V* is a set of communication nodes and sensor nodes random deployed in a 2D sensor field, and *L* is a set of links connect a pair of adjacent communication nodes or between a pair of a sensor node and a communication node.

**Figure 2.**
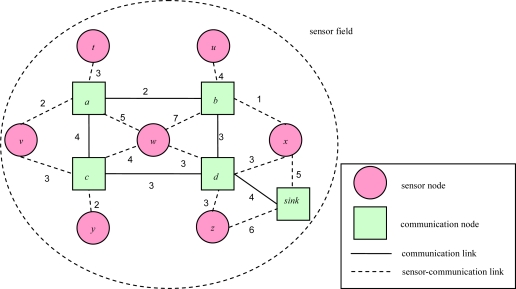
An example of a 2D routing sub-graph.

For example, Figure 2 illustrates a routing sub-graph of a 2D sensor field with each edge connecting a pair of adjacent communication nodes or between a pair of a sensor node and a communication node. Each link weight represents a link transmission cost. In [16], Cartigny, *et al.* defined the energy consumption model of transmitting data which is measured as *r^α^+ c*, where *r* is Euclidean distance between any two nodes, α is a signal attenuation constant, and *c* is a positive constant that represents signal processing. Table 1 presents the power model for the MICAz hardware platform. As the table shows, transmission power and received power are different. To be more generic, we redefine the link transmission cost as the power consumption of transmission power and received power, which is measured as *r^α^*+ *x* + *c*, where *x* is received power.

**Figure 3.**
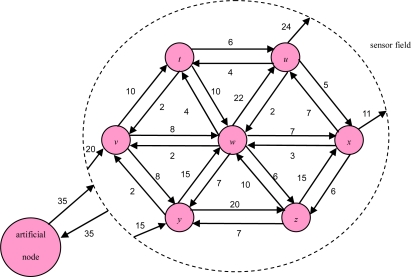
An example of a 2D sensor sub-graph.

The sensor sub-graph in Figure 3 illustrates a 2D sensor field with each edge connecting a pair of adjacent sensors. We use (*i*,*j*) to represent the weight of link which is the moving object frequency of a sensor node *i* and a senor node *j*. The link weight of artificial node is the moving frequency of object between sensor field and outside the sensor field.

**Figure 4.**
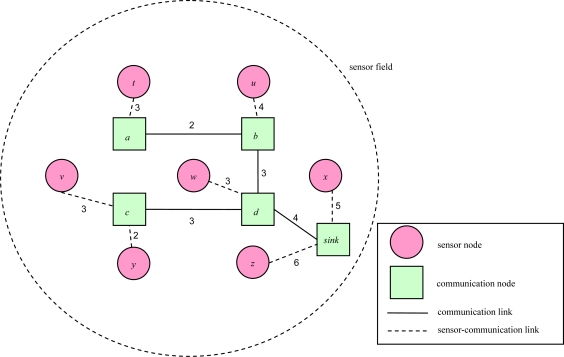
An example of a 2D object tracking tree.

Figure 4 illustrates an object tracking tree of a 2D sensor field with each edge connecting a pair of adjacent nodes. Each link weight represents the link transmission cost between a pair of adjacent communication nodes, or between a pair of a sensor node and a communication node. The root is a sink node.

**Figure 5.**
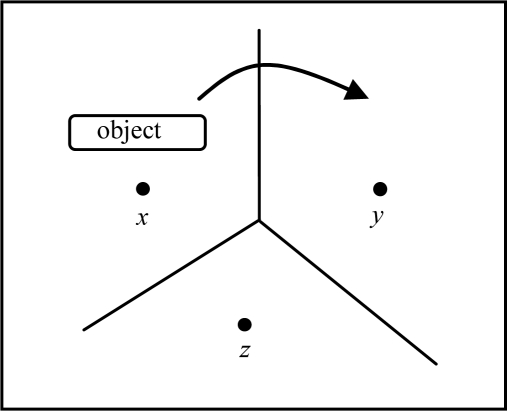
An example of an object moves from voronoi cell *x* to voronoi cell *y*.

In this paper, we consider a given arbitrary topology of sensor networks, bi-directional moving objects with given frequencies for each pair of sensor nodes, and link transmission cost. The sensor field consists of sensor nodes and communication nodes. We deploy hierarchical network topology architecture. All sensor nodes send data to upper layer communication nodes. Eventually, the sensing information is sent to the sink node. We assume that *G* is connected. The location model is a sensor cell model constructed by voronoi diagram. For example, an object moves from sensor *x* to sensor *y* means that the object moves from voronoi cell of sensor *x* to voronoi cell of sensor *y* as shown in Figure 5.

**Figure 6.**
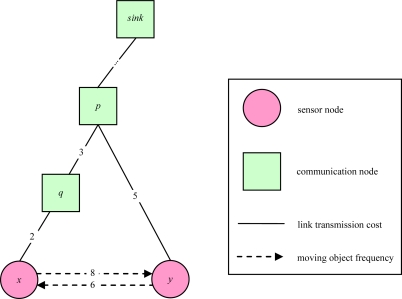
An example of calculating communication cost.

A good tracking method is characterized by a low total communication cost [6]. Given a sensor graph, we can compute the total communication cost. This communication cost calculation is different from that of prior studies [6–8]. First, we consider bi-directional moving objects with given frequencies for each pair of sensor nodes because the round-trip traffic cost of each pair of sensor nodes is different. Second, we consider the link transmission cost since each link transmission cost is also different. Figure 6 illustrates an example of communication cost calculation. The weight of each solid link represents link transmission cost between a pair of adjacent communication nodes or between a pair of a sensor nodes and a communication node. The weight of each dashed link represents the frequency of moving objects between a pair of adjacent sensors. When an object moves from sensor *x* to sensor *y*, sensor *y* needs to deliver the tracking information upward to the nearest common ancestor *p* via the tree links. We call the tree links as the tracking links [6]. For example, the link between communication node *p* and sensor node *y* is a tracking link.

We define the communication cost of an object tracking tree *T* as the sum of the individual contributions of all pairs of sensors adjacent in *G*. Since the adjacent tree nodes may be physically in a distance, we define the costs of tree links used in the path to be Euclidean distances. Thus, the communication cost reflects the power consumption degree of required radio.

The communication cost of inside the network, defined as:

$${\left(G,T\right)}_{\mathrm{inside}}=\sum_{(i,j)\in L}\left(1-z_{\left(i,j\right)}^{x}\right)\;z_{\left(i,j\right)}^{y}r_{\mathrm{xy}}a_{\left(i,j\right)}\;\;\;\;\;\forall x,y\in S.$$

The communication cost of entering the sensor field, defined as:

$${\left(G,T\right)}_{\mathrm{enter}}=\sum_{(i,j)\in L}z_{\left(i,j\right)}^{s}r_{\mathrm{os}}a_{\left(i,j\right)}\;\;\;\;\;\;\forall s\in S.$$

The communication cost of leaving the sensor field, defined as:

$${\left(G,T\right)}_{\mathrm{leave}}=\sum_{(i,j)\in L}z_{\left(i,j\right)}^{s}r_{so}a_{\left(i,j\right)}\;\;\;\;\;\forall s\in S.$$

where *S* is the set of all sensor nodes and *L* is the set of all links. *a*_(*i,j*)_ is the transmission cost associated with link (*i*,*j*). *r_xy_* is the frequency of moving object from *x* to *y*, *r_os_* is the frequency while object enters sensor field, *r_so_* is the frequency when object leaves sensor field, and the tree links, $z_{\left(i,j\right)}^{s}$, are the links of object tracking tree. The decision variable $z_{\left(i,j\right)}^{s}=1$ if the sensor node *s* uses the tree link (*i*,*j*) to reach the sink node, and 0 otherwise.

For example, in Figure 6, communication cost is 5 × 8 = 40 when the object moves from sensor *x* to sensor *y*, and communication cost is (3 + 2) × 6 = 30 when it moves from sensor *y* to sensor *x*. Therefore, the total communication cost for tree *T* as the sum of counting the number of events transmitted in *G*:

$$\text{Total Communication Cost }(G,T)=\sum_{x\in S}\sum_{y\in S}\sum_{(i,j)\in L}(1-z_{\left(i,j\right)}^{x})z_{\left(i,j\right)}^{y}r_{xy}a_{\left(i,j\right)}+\sum_{s\in S}\sum_{(i,j)\in L}z_{\left(i,j\right)}^{s}(r_{\mathrm{os}}+r_{\mathrm{so}})a_{\left(i,j\right)}$$

## 3. Problem Formulation

The notations used to model the problem are listed in Tables 2–4 as follow:

**Table:** 

**Problem (IP1):**
**Objective function:**
(IP1) $$Z_{\mathrm{IP}}=\text{min}\sum_{x\in S}\sum_{y\in S}\sum_{(i,j)\in L}(1-z_{\left(i,j\right)}^{x})z_{\left(i,j\right)}^{y}r_{\mathrm{xy}}a_{\left(i,j\right)}+\sum_{s\in S}\sum_{(i,j)\in L}z_{\left(i,j\right)}^{s}(r_{\mathrm{os}}+r_{\mathrm{so}})a_{\left(i,j\right)}$$
**subject to:**
(1.1) $$\sum_{p\in P_{s}}x_{sp}=1\;\;\;\;\;\;\;\;\;\;\;\;\;\;\;\;\;\;\;\;\forall s\in S$$
(1.2) $$\sum_{{}_{j\in C}}z_{\left(i,j\right)}^{s}=1\;\;\;\;\;\;\;\;\;\;\;\;\;\;\;\;\;\;\;\;\forall s\in S,i\in S\cup C-\{\mathrm{sink}\},i\neq j$$
(1.3) $$\sum_{p\in P_{s}}x_{\mathrm{sp}}\delta_{p(i,j)}\leq z_{\left(i,j\right)}^{s}\;\;\;\;\;\;\;\;\;\;\;\;\;\;\;\;\;\;\;\;\forall s\in S,\;(i,j)\in L,\;i\neq j$$
(1.4) $$\sum_{(i,j)\in L}(1-z_{\left(i,j\right)}^{x})z_{\left(i,j\right)}^{y}\geq 1\;\;\;\;\;\;\;\;\;\;\;\;\;\;\;\;\;\;\;\;\forall x,y\in S,\;x\neq y\;\text{and}\;i\neq j$$
(1.5) $$x_{\mathrm{sp}}=0\;\text{or }1\;\;\;\;\;\;\;\;\;\;\;\;\;\;\;\;\;\;\;\forall s\in S,p\in P_{s}$$
(1.6) $$z_{\left(i,j\right)}^{s}=0\;\text{or }1\;\;\;\;\;\;\;\;\;\;\;\;\;\;\;\;\;\;\;\forall s\in S,\;(i,j)\in L,\text{and}\;i\neq j.$$

The objective function (IP1) of this problem is to minimize the total communication cost subject to:

Constraint (1.1): Routing constraint which uses one path from sensor node *s* to *sink* node only.
Constraint (1.2): Tree constraint of avoiding cycles. Any outgoing link of a node to communication node is equal to 1 on the object tracking tree.
Constraint (1.3): Routing constraint. Once the path, *x_sp_*, is selected and the tree link (*i, j*) is on the path, the decision variable, $z_{\left(i,j\right)}^{s}$, must set to be 1.
Constraint (1.4): Sensor *y* must use one or more tree links (*i*,*j*) to report location of object when object moves from sensor *x* to sensor *y*. Therefore, $\sum_{(i,j)\in L}(1-z_{\left(i,j\right)}^{x})z_{\left(i,j\right)}^{y}$ must be greater than or equal to 1.
Constraints (1.5–1.6): Decision variables *x_sp_* and $z_{\left(i,j\right)}^{s}$ equal to 0 or 1.

Problem (IP1) is hard to solve, since original objective function,

$$\text{min}\sum_{x\in S}\sum_{y\in S}\sum_{(i,j)\in L}(1-z_{\left(i,j\right)}^{x})z_{\left(i,j\right)}^{y}r_{\mathrm{xy}}a_{\left(i,j\right)}+\sum_{s\in S}\sum_{(i,j)\in L}z_{\left(i,j\right)}^{s}(r_{\mathrm{os}}+r_{\mathrm{so}})a_{\left(i,j\right)}$$

and constraint (1.4) are nonlinear.

An auxiliary variable $t_{\left(i,j\right)}^{\mathrm{xy}}$ is introduced. Tracking links, $t_{\left(i,j\right)}^{\mathrm{xy}}$, are the links when object moves from sensor *x* to sensor *y*, and then sensor *y* delivers tracking information upward to the nearest common ancestor via the tracking links, where $t_{\left(i,j\right)}^{\mathrm{xy}}=(1-z_{\left(i,j\right)}^{x})z_{\left(i,j\right)}^{y}$. Table 5 shows the truth table for variables $z_{\left(i,j\right)}^{x}$, $z_{\left(i,j\right)}^{y}$, and $t_{\left(i,j\right)}^{\mathrm{xy}}$.

We also add variable-transformation constraints (2.4 and 2.5) to fulfill the truth table. If $z_{\left(i,j\right)}^{x}=0\cap z_{\left(i,j\right)}^{y}=1$, $t_{\left(i,j\right)}^{\mathrm{xy}}$ must set to be 1, and 0 otherwise.

The constraints can transform nonlinear original objective function to a linear objective function, $\text{min}\sum_{x\in S}\sum_{y\in S}\sum_{(i,j)\in L}t_{\left(i,j\right)}^{\mathrm{xy}}r_{\mathrm{xy}}a_{\left(i,j\right)}+\sum_{s\in S}\sum_{(i,j)\in L}z_{\left(i,j\right)}^{s}(r_{\mathrm{os}}+r_{\mathrm{so}})a_{\left(i,j\right)}$, and linear constraint (2.6).

Therefore, we add the new decision variable, $t_{\left(i,j\right)}^{\mathrm{xy}}$, to reformulate the problem as follows.

**Table:** 

**Problem (IP2):**
**Objective function:**
(IP2) $$Z_{\mathrm{IP}}=\text{min}\sum_{x\in S}\sum_{y\in S}\sum_{(i,j)\in L}t_{\left(i,j\right)}^{\mathrm{xy}}r_{\mathrm{xy}}a_{\left(i,j\right)}+\sum_{s\in S}\sum_{(i,j)\in L}z_{\left(i,j\right)}^{s}(r_{\mathrm{os}}+r_{\mathrm{so}})a_{\left(i,j\right)}$$
**subject to:**
(2.1) $$\sum_{p\in P_{s}}x_{\mathrm{sp}}=1\;\;\;\;\;\;\;\;\;\;\;\;\;\;\;\;\;\;\;\forall s\in S$$
(2.2) $$\sum_{j\in C}z_{\left(i,j\right)}^{s}=1\;\;\;\;\;\;\;\;\;\;\;\;\;\;\;\;\;\;\;\forall s\in S,i\in S\cup C-\{\mathrm{sink}\},i\neq j$$
(2.3) $$\sum_{p\in P_{s}}x_{\mathrm{sp}}\delta_{p(i,j)}\leq z_{\left(i,j\right)}^{s}\;\;\;\;\;\;\;\;\;\;\;\;\;\;\;\;\;\;\;\forall s\in S,(i,j)\in L,i\neq j$$
(2.4) $$2t_{\left(i,j\right)}^{\mathrm{xy}}\leq z_{\left(i,j\right)}^{y}-z_{\left(i,j\right)}^{x}+1\;\;\;\;\;\;\;\;\forall x,y\in S,(i,j)\in L,i\neq j$$
(2.5) $$z_{\left(i,j\right)}^{y}-z_{\left(i,j\right)}^{x}+1\leq t_{\left(i,j\right)}^{\mathrm{xy}}+1\;\;\;\;\;\;\;\;\;\;\;\;\;\;\;\;\;\;\forall x,y\in S,(i,j)\in L,x\neq y\;\text{and }i\neq j$$
(2.6) $$\sum_{(i,j)\in L}t_{\left(i,j\right)}^{\mathrm{xy}}\geq 1\;\;\;\;\;\;\;\;\;\;\;\;\;\;\;\;\;\;\;\;\;\;\;\forall x,y\in S,x\neq y\;\text{and }i\neq j$$
(2.7) $$x_{\mathrm{sp}}=0\;\text{or}\;1\;\;\;\;\;\;\;\;\;\;\;\;\;\;\;\;\;\;\;\;\;\;\forall s\in S,p\in P_{s}$$
(2.8) $$z_{\left(i,j\right)}^{s}=0\;\text{or}\;1\;\;\;\;\;\;\;\;\;\;\;\;\;\;\;\;\;\;\;\;\forall s\in S,(i,j)\in L,\;\text{and}\;i\neq j$$
(2.9) $$t_{\left(i,j\right)}^{\mathrm{xy}}=\;0\;\text{or}\;1\;\;\;\;\;\;\;\;\;\;\;\;\;\;\;\;\;\;\;\;\forall x,y\in S,(i,j)\in L,x\neq y\;\text{and}\;i\neq j$$

The objective function (IP2) of this problem is to minimize the total communication cost subject to:

Constraint (2.1): Routing constraint which uses one path from sensor node *s* to *sink* node only.
Constraint (2.2): Tree constraint of avoiding cycles. Any outgoing link of node to communication node is equal to 1 on the object tracking tree.
Constraint (2.3): Routing constraint. Once the path, *x_sp_*, is selected and the tree link (*i, j*) is on the path, the decision variable, $z_{\left(i,j\right)}^{s}$, must set to be 1.
Constraints (2.4–2.5): There are variable-transformation constraints. If $z_{\left(i,j\right)}^{x}=0\cap z_{\left(i,j\right)}^{y}=1$, reporting location of object will use the tracking link (*i, j*) when object moves from sensor *x* to sensor *y*, $t_{\left(i,j\right)}^{\mathrm{xy}}$ must set to be 1, and 0 otherwise.
Constraint (2.6): Sensor *y* must use one or more tracking link (*i*,*j*) to report object’s location when object moves from sensor *x* to sensor *y*. Therefore, $\sum_{(i,j)\in L}t_{\left(i,j\right)}^{\mathrm{xy}}$ must be greater than or equal to 1.
Constraints (2.7–2.9): Decision variables *x_sp_*, $z_{\left(i,j\right)}^{s}$, and $t_{\left(i,j\right)}^{\mathrm{xy}}$ equal to 0 or 1.

## 4. Solution Approach

### 4.1. Lagrangean Relaxation

**Figure 7.**
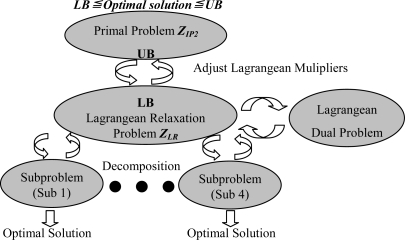
Lagrangean relaxation procedures.

Using the Lagrangean relaxation method, successfully adopted to solve many famous NP-complete problems [14,15], the overall procedure to solve the network planning problem is shown in Figure 7. The relaxation of the primal problem is developed first, which provides a lower bound (LB) on the optimal solutions. Since we relax three constraints of the problem (IP2), the boundary is used to design a heuristic approach to reach a primal feasible solution. To solve the original problem near-optimally and minimize the gap between the primal problem and the Lagrangean dual problem, we improve the LB by solving the four sub-problems optimally and use the subgradient method to adjust the multipliers per iteration. Then, subgradient optimization procedure is used for further improving these solutions by updating the Lagrangean multipliers.

We can transform the primal problem (IP2) into the following Lagrangean relaxation problem (LR) where constraints (2.3), (2.4), and (2.5) are relaxed. For a vector of non-negative Lagrangean multipliers, a Lagrangean relaxation problem of (IP2) is given by:

**Table:** 

**Problem (LR):**
**Objective function:**
(LR) $$\begin{array}{lll} Z_{\mathrm{LR}}(u_{s(i,j)}^{1},u_{\mathrm{xy}(i,j)}^{2},u_{\mathrm{xy}(i,j)}^{3}) & = & \text{min}\{\sum_{x\in S}\sum_{y\in S}\sum_{(i,j)\in L}t_{\left(i,j\right)}^{xy}r_{\mathrm{xy}}a_{\left(i,j\right)}+\sum_{s\in S}\sum_{(i,j)\in L}z_{\left(i,j\right)}^{s}(r_{\mathrm{os}}+r_{\mathrm{so}})a_{\left(i,j\right)}\\ & & +\sum_{s\in S}\sum_{(i,j)\in L}\sum_{p\in P_{s}}u_{s(i,j)}^{1}x_{p}\delta_{p(i,j)}-\sum_{s\in S}\sum_{(i,j)\in L}u_{s(i,j)}^{1}z_{\left(i,j\right)}^{s}\\ & & +\sum_{x\in S}\sum_{y\in S}\sum_{(i,j)\in L}u_{\mathrm{xy}(i,j)}^{2}2t_{\left(i,j\right)}^{\mathrm{xy}}-\sum_{x\in S}\sum_{y\in S}\sum_{(i,j)\in L}u_{\mathrm{xy}(i,j)}^{2}(z_{\left(i,j\right)}^{y}-z_{\left(i,j\right)}^{x}+1)\\ & & +\sum_{x\in S}\sum_{y\in S}\sum_{(i,j)\in L}u_{\mathrm{xy}(i,j)}^{3}(z_{\left(i,j\right)}^{y}-z_{\left(i,j\right)}^{x}+1)-\sum_{x\in S}\sum_{y\in S}\sum_{(i,j)\in L}u_{\mathrm{xy}(i,j)}^{3}(t_{\left(i,j\right)}^{\mathrm{xy}}+1)\} \end{array}$$
**subject to:** (2.1), (2.2), (2.6), (2.7), (2.8), and (2.9).

Where $u_{s(i,j)}^{1}$, $u_{\mathrm{xy}(i,j)}^{2}$, and $u_{\mathrm{xy}(i,j)}^{3}$ are Lagrangean multipliers and $u_{s(i,j)}^{1}$, $u_{\mathrm{xy}(i,j)}^{2}$, and $u_{\mathrm{xy}(i,j)}^{3}\geq 0$. To solve (LR), we can decompose (LR) into the following four independent and easily solvable optimization sub-problems.

$$Z_{\mathrm{LR}}=Z_{\mathrm{sub}1}+Z_{\mathrm{sub}2}+Z_{\mathrm{sub}3}+Z_{\mathrm{sub}4}$$

**Table:** 

**Sub-problem 1: (related to the decision variables**$t_{\left(i,j\right)}^{\mathrm{xy}}$**)**
**Objective function:**
(sub 1) $$Z_{\mathrm{sub}1}(u_{\mathrm{xy}(i,j)}^{2},u_{\mathrm{xy}(i,j)}^{3})=\text{min}\sum_{x\in S}\sum_{y\in S}\sum_{(i,j)\in L}t_{\left(i,j\right)}^{\mathrm{xy}}(r_{\mathrm{xy}}a_{\left(i,j\right)}+2u_{\mathrm{xy}(i,j)}^{2}-u_{\mathrm{xy}(i,j)}^{3})$$
**subject to:** (2.6) and (2.9).

Sub-problem 1 is related to decision variable $t_{\left(i,j\right)}^{\mathrm{xy}}$, which can be further decomposed into |*S*|^2^ |*L*| sub-problems.

Two cases are listed below to determine the value of $t_{\left(i,j\right)}^{\mathrm{xy}}$.

Let *θ*_*xy*(*i,j*)_ denote the weight of the object while moving from sensor *x* to sensor *y* using the tracking link (*i*, *j*), we get

$$\theta_{\mathrm{xy}(i,j)}=(r_{\mathrm{xy}}a_{\left(i,j\right)}+2u_{\mathrm{xy}(i,j)}^{2}-u_{\mathrm{xy}(i,j)}^{3})$$

Case 1: If *θ*_*xy*(*i,j*)_< 0, then assign $t_{\left(i,j\right)}^{\mathrm{xy}}=1$

Case 2: If *θ*_*xy*(*i,j*)_ ≥ 0, then assign $t_{\left(i,j\right)}^{\mathrm{xy}}=0$.

If the sum of each pair of node $t_{\left(i,j\right)}^{\mathrm{xy}}$ is zero, we enforce to select the minimum positive objective value *θ*_*xy*(*i,j*)_ and set $t_{\left(i,j\right)}^{\mathrm{xy}}=1$ to fulfill the constraint (6). The time complexity of the sub-problem is *O*(|*S*|^2^|*L*|).

**Table:** 

**Sub-problem 2: (related to the decision variables *x****_sp_***)**
**Objective function:**
(sub 2) $$Z_{\mathrm{sub}2}\;(u_{s(i,j)}^{1})=\text{min}\sum_{s\in S}\sum_{(i,j)\in L}(u_{s(i,j)}^{1}\sum_{p\in P}x_{\mathrm{sp}}\delta_{p(i,j)})$$
**subject to:** (2.1) and (2.7).

Sub-problem 2 can be further decomposed into |*S*| independent shortest path problems with nonnegative arc weight whose value is $u_{s(i,j)}^{1}$. The value of *x_sp_* can be determined by the link cost, $u_{s(i,j)}^{1}$. This sub-problem is related to the decision variables *x_sp_*, which can use the Dijkstra’s algorithm to solve the single source shortest path problem. The time complexity of Dijkstra’s algorithm is *O*(|*S*|^2^). The time complexity of the sub-problem is *O*(|*S*|^3^).

**Table:** 

**Sub-problem 3: (related to the decision variables**$z_{\left(i,j\right)}^{s}$**)**
**Objective function:**
(sub 3) $$Z_{\mathrm{sub}3}\;(u_{s(i,j)}^{1},u_{\mathrm{xy}(i,j)}^{2},u_{\mathrm{xy}(i,j)}^{3})=\text{min}\sum_{s\in S}\sum_{(i,j)\in L}[(r_{\mathrm{os}}+r_{\mathrm{so}})a_{\left(i,j\right)}-u_{s(i,j)}^{1}+\sum_{x\in S}(u_{\mathrm{xs}(i,j)}^{3}-u_{\mathrm{xs}(i,j)}^{2})-\sum_{y\in S}(u_{\mathrm{sy}(i,j)}^{3}-u_{sy(i,j)}^{2})]z_{\left(i,j\right)}^{s}$$
**subject to:** (2.2) and (2.8).

Sub-problem 3 is related to the decision variables $z_{\left(i,j\right)}^{s}$ which can be further decomposed into |*S*| sub-problems.

Let *ψ*_*s*(*i, j*)_ denote the weight of the sensor nodes s using the tree link (*i*, *j*), we get

$$\psi_{s(i,j)}=(r_{\mathrm{os}}+r_{\mathrm{so}})a_{\left(i,j\right)}-u_{s(i,j)}^{1}+\sum_{x\in S}(u_{\mathrm{xs}(i,j)}^{3}-u_{\mathrm{xs}(i,j)}^{2})-\sum_{y\in S}(u_{\mathrm{sy}(i,j)}^{3}-u_{\mathrm{sy}(i,j)}^{2})$$

$z_{\left(i,j\right)}^{s}$ must be enforced to 1 when choosing the minimum of *ψ*_*s*(*i, j*)_ for each *s* and *i*_to fulfill the constraint (2.2). The time complexity of this sub-problem is *O*(|*S*|^2^|*L*|).

**Table:** 

**Sub-problem 4: (Constant Part)**
**Objective function:**
(sub 4) $$Z_{\mathrm{sub}4}\;(u_{\mathrm{xy}(i,j)}^{2})=-\sum_{x\in S}\sum_{y\in S}\sum_{(i,j)\in L}u_{\mathrm{xy}(i,j)}^{2}$$

Sub-problem 4 is a constant part. The time complexity of the sub-problem is *O*(|*S*|^2^|*L*|).

According to the weak Lagrangean duality theorem [14,15], $Z_{D}(u_{s(i,j)}^{1},u_{\mathrm{xy}(i,j)}^{2},u_{\mathrm{xy}(i,j)}^{3})$ is a lower bound (LB) on *Z_IP_* when $u_{s(i,j)}^{1}$, $u_{\mathrm{xy}(i,j)}^{2}$, and $u_{\mathrm{xy}(i,j)}^{3}\geq 0$. The following dual problem (D) is then constructed to calculate the tightest lower bound.

**Table:** 

**Dual Problem (D):**
**Objective function:**
(D) $$Z_{D}=\text{max}\;Z_{\mathrm{LR}}\;(u_{s(i,j)}^{1},u_{xy(i,j)}^{2},u_{\mathrm{xy}(i,j)}^{3})$$
**subject to:**
(2.10) $$u_{s(i,j)}^{1},u_{\mathrm{xy}(i,j)}^{2},\;\text{and}\;u_{\mathrm{xy}(i,j)}^{3}>0$$

There are several methods for solving the dual-mode problem (D). One of the most popular approaches is the subgradient method.

### 4.2. Getting Primal Feasible Solutions

**Figure 8.**
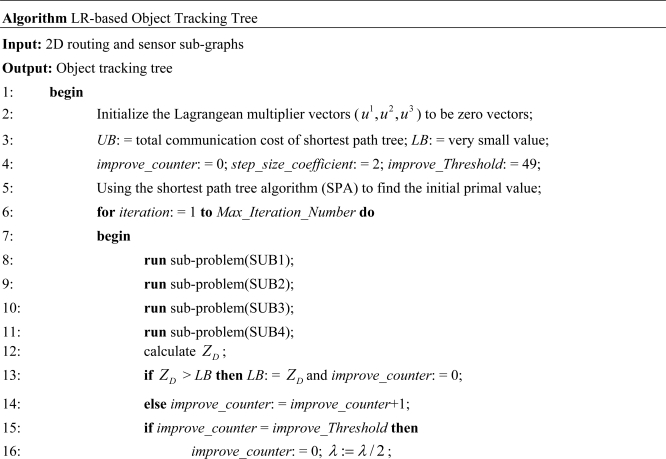

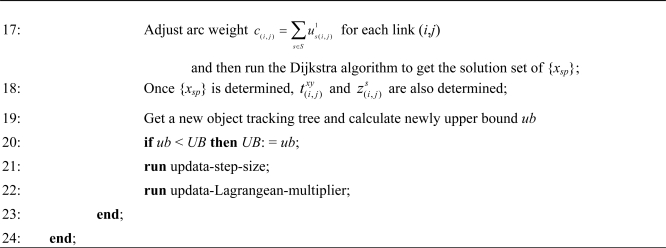
The LR-based object tracking tree algorithm.

After optimally solving the Lagrangean dual problem, we obtain a set of decision variables and develop a LR-based heuristic algorithm to tune these decision variables. A set of feasible solutions of the primal problem (IP2) can therefore be obtained. The primal feasible solution is an upper bound (*UB*) of the primal problem (IP2), and the Lagrangean dual problem solution guarantees the lower bound (*LB*) of the primal problem (IP2). Iteratively, by solving primal feasible solution and Lagrangean dual problem, we get *UB* and *LB*, respectively. A LR-based primal heuristic object tracking tree algorithm is listed in Figure 8.

In the algorithm, from Steps 2–4 are setting the initialize value, Step 5 is finding the initial primal value. Steps 8–11 solve the sub-problems 1–4. Steps 12–16 and 20–22 update the parameters and multipliers. Steps 17–19 are used to get primal feasible solution.

## 5. Computational Experiments

To evaluate the performance of the proposed algorithm, we conducted an experiment. The performance is assessed in terms of the total communication cost.

### 5.1. Experiments Environment

The proposed algorithm is coded in C++ under a Microsoft Visual C++ 6.0 development environment. All the experiments are performed on a Core 2 Duo-2.2 GHz PC with 4GB memory running Microsoft Windows VISTA. The algorithm is tested on a 2D sensor field. We distribute 12, 23, 36, 50, and 105 sensor and communication nodes, respectively, in a 2D sensor field. The parameters listed in Table 7 are used for the all cases of experiments.

### 5.2. Experimental Results

**Figure 9.**
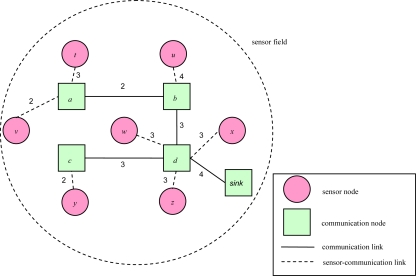
Example of an LR-based object tracking tree.

In order to evaluate the proposed LR-based algorithm, we compare the algorithm with another heuristic algorithm, the shortest path tree (SPT) algorithm. We also compare the proposed LR-based algorithm with the lower bound (*LB*) of the dual mode problem. Figure 9 shows an example of the LR-based object tracking tree.

Table 8 shows the total communication cost calculated by different algorithms under the scenarios of number of nodes = 12, 23, 36, 50, and 105, respectively. We can see that the LR-based heuristic algorithm outperforms the SPT algorithm. We denote the dual solution as “*Z_du_*” (*LB*), and LR-based heuristic solution as “*Z_IP2_*” (*UB*). The gap between *UB* and *LB* is computed by |(UB − LB)/LB| × 100% which illustrates the optimality of problem solution.

**Figure 10.**
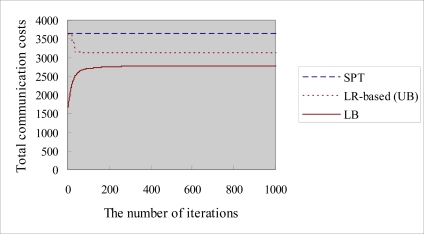
The execution results of LR-based algorithm with 12 nodes in test problem 1.

Figure 10 shows an example of the trend line for getting the primal problem solution values (*UB*) and dual mode problem values (*LB*). The *UB* curve tends to decrease to reach the minimum feasible solution. In contrast, the *LB* curve tends to increase and converge rapidly to reach the optimal solution. The LR-based method ensures the optimization results between *UB* and *LB* so that we can keep the duality gap as small as possible in order to improve the quality of the solution and achieve near optimization.

Table 9 shows that the time complexity of the LR-based solution is dominated by the Lagrangean dual problem. The Lagrangean dual problem has been solved by the above four sub-problems with the maximum number of iteration *I*.

## 6. Conclusions

This study proposes an object tracking algorithm for wireless sensor networks. To our best knowledge, the proposed algorithm is truly novel and it has not been discussed in previous research. This study first formulates the problem as a 0/1 integer programming problem, and then proposes a Lagrangean relaxation-based heuristic algorithm to solve the optimization problem. The experimental results show that the algorithm is better than the shortest path tree algorithm, and the gap is also small. In other words, when compared with the shortest path tree algorithm, the proposed heuristic algorithm can improve the percentage of energy consumption from 8% to 44%. It also achieves the near optimal solution since the gaps are only from 8% to 18%. Therefore, the results show that the proposed Lagrangean relaxation-based algorithm can achieve energy-efficient object tracking. Furthermore, the algorithm is very efficient and scalable in terms of the solution time. We are planning to further investigate a response time model based on object tracking application requirements and heuristic algorithms in the near future. In addition, we are looking into the tradeoff between total communication cost and various other system issues, such as response time, report frequency, and number of sinks, etc.

## Figures and Tables

**Table 1.** Power model of the MICAz.

**Mode**		**Current**
Radio	Rx	19.7 mA
	Tx(−10 dBm)	11 mA
	Tx(−5 dBm)	14 mA
	Tx(0 dBm)	17.4 mA

**Table 2.** Notations for the given parameters.

**Given Parameters**	
Notation	Description
*S*	The set of all sensor nodes.
*C*	The set of all communication nodes, including sink node.
*o*	Artificial node outside the sensor field.
*R*	The set of the object moving frequency from *x* to *y*, ∀*x, y* ∈ *S* ∪ {*o*}, *x* ≠ *y*.
*L*	The set of all links, (*i, j*)∈ *L*, *i*≠*j*.
*A*	The set of transmission costs *a_(i,j)_* associated with link (*i, j*).
*P_s_*	The set of all candidate paths *p* between a pair of nodes, *s and* sink, ∀*s* ∈ *S*.

**Table 3.** Notation for the indicate parameter.

**Indicate Parameter**	
Notation	Description
*δ_p(i,j)_*	The value of indicator function is 1 if link (*i, j*) is on path *p*, and 0 otherwise.

**Table 4.** Notations for the decision variables.

**Decision Variables**	
Notation	Description
*x_sp_*	1 if the sensor node *s* uses the path *p* to reach the sink node, and 0 otherwise.
$z_{\left(i,j\right)}^{s}$	1 if the sensor node *s* uses the link (*i, j*) to reach the sink node, and 0 otherwise.

**Table 5.** The truth table of variables $z_{\left(i,j\right)}^{x}$, $z_{\left(i,j\right)}^{y}$, and $t_{\left(i,j\right)}^{\mathrm{xy}}$.

$z_{\left(i,j\right)}^{x}$	$z_{\left(i,j\right)}^{y}$	$t_{\left(i,j\right)}^{\mathrm{xy}}$
0	0	0
0	1	1
1	0	0
1	1	0

**Table 6.** Notations for the decision variable $t_{\left(i,j\right)}^{\mathrm{xy}}$.

**Decision Variables**	
Notation	Description
$t_{\left(i,j\right)}^{\mathrm{xy}}$	1 if $z_{\left(i,j\right)}^{x}=0\cap z_{\left(i,j\right)}^{y}=1$ (reporting object’s location uses the link (*i,j*) when object moves from sensor *x* to sensor *y*), and 0 otherwise, *x* ≠ *y*.

**Table 7.** Parameter of Lagrangean relaxation-based algorithm.

**Parameter**	**Value**

Number of nodes	12–105 (depend on each case)
Number of iterations	5,000
Improvement counter threshold	49
Initial upper bound	10^10^
Initial lower bound	−10^10^
Initial scalar of step size	2
Initial multiplier	0

**Table 8.** Evaluation of the gap and improvement ratio with different number of nodes.

**Number of nodes**		***Z_du_***	***Z_IP2_***	**Gap**	**SPT**	**Improvement Ratio to SPT**
12	Problem 1 Problem 2	2,774 3,416	3,127 3,906	0.13 0.14	3,630 4,460	0.16 0.14
23	Problem 1 Problem 2	17,850 17,385	20,725 20,282	0.16 0.17	22,491 21,839	0.09 0.08
36	Problem 1 Problem 2	42,410 42,775	49,970 50,411	0.18 0.18	57,553 57,787	0.15 0.15
50	Problem 1 Problem 2	89,824 77,905	78,807 88,195	0.14 0.13	99,639 102,796	0.11 0.17
105	Problem 1 Problem 2	326,529 328,911	371,438 355,546	0.14 0.08	508,314 511,402	0.37 0.44

**Table 9.** The time complexity of LR-based object tracking tree algorithm.

**Problem**	**Time Complexity**

Sub-problem (SUB1)	*O*(|*S*|^2^|*L*|)
Sub-problem (SUB2)	*O*(|*S*|^3^)
Sub-problem (SUB3)	*O*(|*S*|^2^|*L*|)
Sub-problem (SUB4)	*O*(|*S*|^2^|*L*|)
Getting primal feasible solutions	*O*(|*S*|^2^)
Lagrangean dual problem	*O*(*I*|*S*|^2^|*L*|)^*^

* Parameter *I* means the maximum number of iterations
